# Heterozygous Hemoglobin Sherwood Forest Causing Polycythemia

**DOI:** 10.1155/2017/8174207

**Published:** 2017-09-28

**Authors:** Vikram M. Raghunathan, James N. Butera, Diana O. Treaba

**Affiliations:** ^1^Department of Medicine, Brown University Alpert Medical School, Providence, RI, USA; ^2^Division of Hematology and Oncology, Rhode Island Hospital, Providence, RI, USA; ^3^Department of Pathology and Laboratory Medicine, Brown University Alpert Medical School, Providence, RI, USA

## Abstract

Hemoglobin (Hb) Sherwood Forest is a rare high-affinity hemoglobin first described in 1977, arising from an Arg to Thr substitution at codon 104 of the beta chain. This hemoglobin variant has been identified in few individuals and has been associated with a compensatory erythrocytosis in the homozygous state. Prior scarce case reports have noted that heterozygotes for this variant are phenotypically normal. Here we present a patient who was evaluated in our hematology clinic for chronic erythrocytosis and was found to be heterozygous for Hb Sherwood Forest. No other primary or secondary cause of his polycythemia was identified. This is the first described case of heterozygous Hemoglobin Sherwood Forest causing erythrocytosis.

## 1. Case

A 50-year-old man with atrial fibrillation, subaortic stenosis, and hyperthyroidism presented to hematology clinic for evaluation of longstanding erythrocytosis. He was asymptomatic and had not experienced any episodes of thrombosis or hyperviscosity in the past. He was referred for work-up of chronically elevated hematocrit by his primary care provider. The patient had no history of chronic lung disease, obstructive sleep apnea, tobacco use, renal disease, or use of exogenous androgens or erythropoietin. He stated that his siblings in Iran also had erythrocytosis requiring serial phlebotomy.

**Figure 1:**
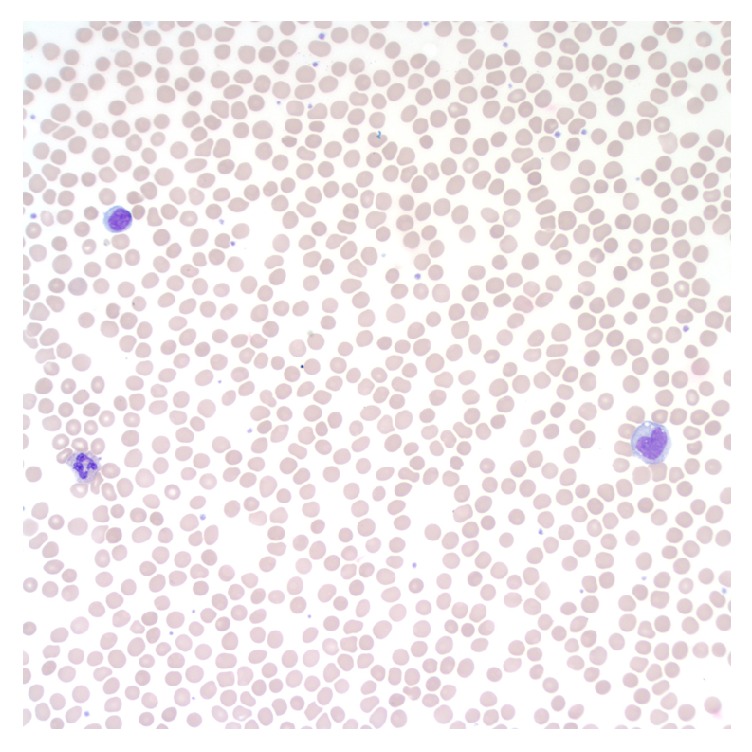
Peripheral blood smear with normocytic normochromic red blood cells. Wright stain, immersion oil 50x.

On initial evaluation the patient was hemodynamically stable without hypoxia. His physical exam was unremarkable, without splenomegaly or other findings suggestive of a hematologic disorder. His hemoglobin level was 19.5 g/dL with a hematocrit of 58 percent. Peripheral blood smear was unrevealing (Figure 1). His serum erythropoietin level, at 11.8 mIU/mL, was inappropriately high for his degree of polycythemia. Methemoglobin level was 0.5 percent. JAK2 V617F mutation testing was negative, effectively excluding polycythemia vera. A p50 red blood cell oxygen dissociation assay showed a left-shifted curve with a venous p50 of 23 mm Hg, suggestive of a high oxygen-affinity hemoglobin variant. In the setting of this finding, JAK2 exon 12 mutation testing was not pursued as it would not explain his elevated oxygen affinity, nor was bone marrow biopsy performed.

**Figure 2:**
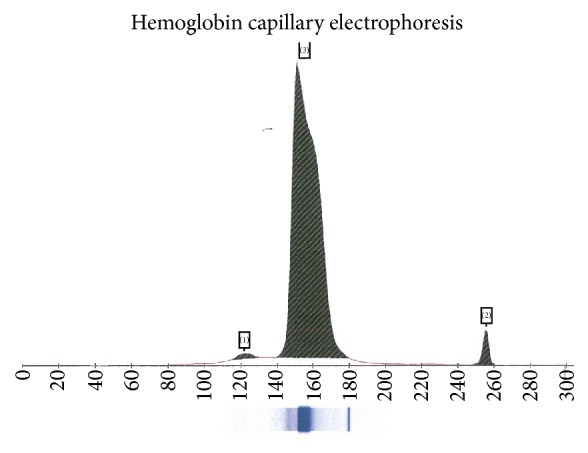
Sebia hemoglobin capillary electrophoresis. Fraction (1): hemoglobin F 0.7%; Fraction (2): hemoglobin A2 2.9%; Fraction (3): beta globin variant and closely located Hemoglobin A: 96.4%.

Hemoglobin electrophoresis showed a beta globin variant migrating very close to Hb A; an acid gel revealed that Hb A and this variant occurred in nearly equal proportions (Figure 2). Subsequent hemoglobin electrophoresis cascade revealed a 48.9 percent share of Hb A and a 48.3 percent share of the variant hemoglobin, which was identified as Hb Sherwood Forest by molecular bidirectional sequence analysis to test for the presence of a mutation in all coding regions and noncoding portions of the beta hemoglobin gene. As no other etiology for his erythrocytosis had been identified, the patient's condition was believed to be caused by his heterozygous Hb Sherwood Forest. He did not require any treatment for his polycythemia and continues to be monitored on an outpatient basis.

## 2. Discussion

High-affinity hemoglobinopathies are a relatively rare cause of erythrocytosis, and their clinical effects may be mistaken for more common conditions, such as polycythemia vera or erythrocytosis of chronic lung disease. The diagnosis of high-affinity hemoglobin variants typically requires a p50 oxygen dissociation assay. On the hemoglobin-oxygen dissociation curve, p50 refers to the partial pressure of oxygen at which the hemoglobin molecule is half-saturated with oxygen. A high-affinity hemoglobin variant will have a left-shifted oxygen dissociation curve, reflecting the aberrant molecule's ability to bind oxygen at relatively low oxygen pressure. The resulting impairment in oxygen release causes relative tissue hypoxia, stimulating production of erythropoietin and increased red blood cell mass and polycythemia [1].

Many high-affinity hemoglobin variants have been identified, with mutations affecting numerous sites on the alpha or beta chain. All high-affinity hemoglobin mutations favor the relaxed (“R”) state, the molecular conformation that preferentially binds ligands such as oxygen and carbon monoxide [2]. The earliest high-affinity hemoglobin to be described was Hb Chesapeake, caused by a Leu to Arg substitution on the alpha chain first identified in 1966 [3]. Since then, nearly 200 high-affinity hemoglobin variants have been discovered, nearly all caused by single amino acid substitutions [4].

Investigation of our patient's erythrocytosis led to a diagnosis of Hb Sherwood Forest, a high-affinity hemoglobin that has been infrequently described in the medical literature. Ryrie et al. first identified Hb Sherwood Forest in 1977 in a young woman with anemia during pregnancy [5]. Hemoglobin electrophoresis revealed a variant migrating in close proximity to Hb A; the aberrant molecule was found to have an Arg to Thr substitution in codon 104 of the beta chain. This heterozygous patient did not appear to have any clinical manifestation of her abnormal hemoglobin, and her anemia improved after delivery. The investigators concluded that the amino acid substitution had no effect on the function of the hemoglobin molecule.

Nearly two decades later, Williamson et al. described a young man with polycythemia who was found to be homozygous for the same high-affinity hemoglobin [6]. This was the first reported case of homozygous Hb Sherwood Forest, and it showed that homozygotes had impaired oxygen delivery with a compensatory erythrocytosis. This individual's parents were both discovered to be heterozygotes for the mutation and had no hematologic abnormality.

Subsequent studies by Schnedl et al. and others showed that Hb Sherwood Forest could result in abnormally elevated Hb A1c values as determined by high-performance liquid chromatography [7, 8]. However, the variant in the heterozygous state was felt to be clinically silent, particularly in light of prior reports of phenotypically normal heterozygotes. Of note, certain other high-affinity hemoglobin molecules have been associated with erythrocytosis in heterozygotes; for instance, a single nucleotide polymorphism at *β*99 produces a hemoglobinopathy that results in a milder erythrocytosis when cooccurring with normal hemoglobin [9]. No such evidence existed for Hb Sherwood Forest and its clinical implications in the heterozygous state were not further investigated. The majority of reported cases of Hb Sherwood Forest occurred in Pakistan and Iran, our patient's region of origin, and this may have contributed to limited documentation of heterozygotes in the medical literature.

In the evaluation of a patient with polycythemia, the identification of high-affinity hemoglobinopathies can prevent unnecessary testing and treatment. Patients with these congenital conditions typically do not require phlebotomy and should not be exposed to cytotoxic treatments used for other conditions, such as polycythemia vera [10]. Based on our patient's clinical experience, we feel that there is compelling evidence that Hb Sherwood Forest in the heterozygous state can cause erythrocytosis. As our understanding of the transmission and expression of high-affinity hemoglobinopathies expands, and previously underrepresented patient populations are further studied, we will develop greater insight into the implications of this family of hematologic conditions.
